# A focus on intra-abdominal infections

**DOI:** 10.1186/1749-7922-5-9

**Published:** 2010-03-19

**Authors:** Massimo Sartelli

**Affiliations:** 1 Department of Surgery, Macerata Hospital - Via Santa Lucia 2, 62100 Macerata - Italy

## Abstract

Complicated intra-abdominal infections are an important cause of morbidity and are frequently associated with poor prognosis, particularly in higher risk patients.

Well defined evidence-based recommendations for intra-abdominal infections treatment are partially lacking because of the limited number of randomized-controlled trials.

Factors consistently associated with poor outcomes in patients with intra-abdominal infections include increased illness severity, failed source control, inadequate empiric antimicrobial therapy and healthcare-acquired infection.

Early prognostic evaluation of complicated intra-abdominal infections is important to select high-risk patients for more aggressive therapeutic procedures.

The cornerstones in the management of complicated intra-abdominal infections are both source control and antibiotic therapy.

The timing and the adequacy of source control are the most important issues in the management of intra-abdominal infections, because inadequate and late control of septic source may have a negative effect on the outcomes.

Recent advances in interventional and more aggressive techniques could significantly decrease the morbidity and mortality of physiologically severe complicated intra-abdominal infections, even if these are still being debated and are yet not validated by limited prospective trials.

Empiric antimicrobial therapy is nevertheless important in the overall management of intra-abdominal infections. Inappropriate antibiotic therapy may result in poor patient outcomes and in the appearance of bacterial resistance.

Antimicrobial management is generally standardised and many regimens, either with monotherapy or combination therapy, have proven their efficacy. Routine coverage especially against Enterococci and candida spp is not always recommended, but can be useful in particular clinical conditions. A de escalation approach may be recommended in patients with specific risk factors for multidrug resistant infections such as immunodeficiency and prolonged antibacterial exposure.

Therapy should focus on the obtainment of adequate source control and adequate use of antimicrobial therapy dictated by individual patient risk factors. Other critical issues remain debated and more controversies are still open mainly because of the limited number of randomized controlled trials.

## Introduction

Intra-abdominal infections (IAI) include many pathological conditions, ranging from uncomplicated appendicitis to faecal peritonitis. IAI are classified into uncomplicated and complicated [1].

In uncomplicated IAIs the infectious process only involves a single organ and does not proceed to peritoneum. Patients with such infections can be managed with either surgical resection alone, or with antibiotics alone. When the focus of infection is treated effectively by surgical excision, 24 hours perioperative prophylaxis is sufficient. Patients with intra-abdominal infection, including acute diverticulitis and certain forms of acute appendicitis, may be managed nonoperatively.

In complicated IAIs, the infectious process proceeds beyond the organ, and causes either localized peritonitis or diffuse peritonitis. The treatment of patients with complicated intra-abdominal infections involves both source control and antibiotic therapy.

Complicated intra-abdominal infections represent an important cause of morbidity and are frequently associated with poor prognosis.

Peritonitis is classified into primary, secondary or tertiary peritonitis [2].

Primary peritonitis is a diffuse bacterial infection without loss of integrity of the gastrointestinal tract. It is rare. It mainly occurs in infancy and early childhood and in cirrhotic patients.

Secondary peritonitis, the most common form of peritonitis, is an acute peritoneal infection resulting from loss of integrity of the gastrointestinal tract or from infected viscera. It is caused by perforation of the gastrointestinal tract (e.g. perforated duodenal ulcer) by direct invasion from infected intra-abdominal viscera (e.g. gangrenous appendicitis). Anastomotic dehiscences are common causes of peritonitis in the postoperative period.

Tertiary peritonitis is a recurrent infection of the peritoneal cavity that follows either primary or secondary peritonitis.

Mortality rates associated with secondary peritonitis with severe sepsis or septic shock have reported an average mortality of approximately 30% [3-5].

Intra-abdominal infections are also classified into community-acquired intra-abdominal infections (CA-IAIs) and healthcare-acquired intra-abdominal infections (HA-IAIs). CA-IAIs are acquired in community, HA-IAIs develop in hospitalized patients or residents of long-term care facilities. They are characterized by increased mortality because of both underlying patient health status and increased likelihood of infection caused by multi drugs resistant organisms [6].

## Prognostic evaluation

Early prognostic evaluation of complicated intra-abdominal infections is important to assess the severity and the prognosis of the disease.

Factors influencing the prognosis of patients with complicated intra-abdominal infections include advanced age, poor nutrition, pre-existing diseases, immunodepression, extended peritonitis, occurrence of septic shock, poor source control, organ failures, prolonged hospitalization before therapy, and infection with nosocomial pathogens [7-14].

Scoring systems can be broadly divided into two groups: disease-independent scores for evaluation of serious patients requiring care in the intensive care unit (ICU) such as APACHE II and Simplified Acute Physiology Score (SAPS II) and peritonitis-specific scores such as MPI [8].

Although previously considered a good marker, APACHE II value in peritonitis has been questioned because of the APACHE II impossibility to evaluate interventions, despite the fact that interventions might significantly alter many of the physiological variables [15]. The MPI is specific for peritonitis and easy to calculate, even during surgery. MPI is calculated using simple factors such as degree of peritonitis, age, sex, time from perforation to operation, origin of sepsis, kind of exudates (clear, purulent or faecal).

Billing et al. [16] demonstrated the reliability of MPI in 2003 patients from 7 centres in Europe. With a threshold index score of 26, the sensitivity was 86 (range 54-98) per cent, specificity 74 (range 58-97) per cent and accuracy 83 (range 70-94) per cent in predicting death. For patients with a score less than 21 the mean mortality rate was 2.3 (range 0-11) per cent, for score 21-29 22.5 (range 10.6-50) per cent and for score greater than 29 59.1 (range 41-87) per cent. In this study the Mannheim peritonitis index provided an easy and reliable means of risk evaluation and classification for patients with peritoneal inflammation.

In 2008 Panhofer et al. [17] published a retrospective single-centre cohort study in patients who developed tertiary peritonitis, proposing a combination of both MPI and APACHE II, concluding that combination of prognostic scores was very useful to detect tertiary peritonitis.

Hypothesizing that intrinsic risk factors were a better predictor of mortality rather than the type of infection, Inui at al. [18] recently investigated the utility of Charlson Comorbidity Index and multiple organ dysfunction (MOD). They reviewed retrospectively 452 patients with IAI who had been treated over 8 years (June 1999-June 2007). Charlson Comorbidity Index and Multiple Organ Dysfunction (MOD) scores were evaluated at admission and on postoperative day 7. When patients with appendicitis were excluded, there was no difference in mortality or complications between patients with CA-IAI and HA-IAI. Statistical analysis demonstrated that catheter-related bloodstream infection, cardiac event, and age > or = 65 were independent risk factors for mortality. Among patients who failed initial therapy, a non-appendiceal source of infection and a Charlson score > or = 2 were determined to be independent risk factors. Non-appendiceal source of infection and MOD score > or = 4 on postoperative day 7 were found to be independent predictors for re-intervention.

## Diagnosis

In the patient with abdominal sepsis early detection and treatment is essential to minimize complications [19].

Complicated intra-abdominal infections diagnosis is mainly a clinical diagnosis.

Abdominal pain, which may be acute or insidious. Initially, the pain may be dull and poorly localized (visceral peritoneum) and often progresses to steady, severe, and more localized pain (parietal peritoneum).

Systemic manifestations are SIRS manifestations: Core body temperature > 38°C or < 36°C, heart rate > 90 beats per minute, respiratory rate > 20 breaths per minute (not ventilated) or PaCO2 < 32 mm Hg (ventilated), WBC > 12,000, < 4,000 or > 10% immature forms (bands) [20].

Hypotension and hypoperfusion signs such as lactic acidosis, oliguria, and acute alteration of mental status are indicative of evolution to severe sepsis.

Abdominal rigidity suggests peritonitis and the need for urgent laparotomy.

The diagnostic approach to confirm abdominal infection source in septic patients depends on the hemo-dynamic stability of the patient.

Unstable Patients may not perform studies that require trips away from the ICU or emergency department [19]. In these patients intra-abdominal septic source may be detected by ultrasound (US).

Abdominal ultrasound, that has the advantage of being portable, may be helpful in the evaluation of right upper quadrant (e.g. perihepatic abscess, cholecystitis, pancreatitis), right lower quadrant, and pelvic pathology (e.g. appendicitis, tubo-ovarian abscess, Douglas abscess), but the examination is sometimes limited because of patient discomfort, abdominal distension, and bowel gas interference [21].

When patients are stable, computerized tomography (CT) is the imaging modality of choice for most intra-abdominal processes [22].

Computed tomography (CT) of the abdomen and the pelvis, when it is possible to perform it, remains the diagnostic study of choice for intra-abdominal infections. CT can detect small quantities of fluid, areas of inflammation, and other GI tract pathology, with a very high sensitivity [23].

The value of both CT and US in the diagnostic work-up for intra-abdominal infections has been fully studied in relation to acute appendicitis. A meta-analysis by Doria et al. [24] evaluated the diagnostic performance of ultrasonography (US) and computed tomography (CT) for the diagnosis of appendicitis in pediatric and adult populations. This meta-analysis found that pooled sensitivity and specificity for diagnosis of appendicitis in children were 88% and 94%, respectively, for ultrasound studies and 94% and 95%, respectively, for CT studies. Pooled sensitivity and specificity for diagnosis in adults were 83% and 93%, respectively, for ultrasound studies and 94% and 94%, respectively, for CT studies.

From the diagnostic performance perspective, CT has a significantly higher sensitivity than US in studies of children and adults; from the safety perspective, however, the radiation associated with CT, especially in children, should be always considered.

An option in the diagnosis of critically ill patients in ICU is bedside diagnostic laparoscopy. It avoids patient transport, is may be very accurate, and maintains ICU monitoring. Bedside diagnostic laparoscopy for intraabdominal diseases has high diagnostic accuracy and in unstable patients with abdominal sepsis of unknown origin, it may be regarded as a good diagnostic [25].

Laparoscopy is gaining wider acceptance in emergency surgery [26]. Diagnostic laparoscopy is widely used to identify the causative pathology of acute abdominal pain. It may also be followed by laparoscopic treatment of the detected abdominal disorder [27,28].

The accuracy of diagnostic laparoscopy is very high. In the last years studies have reported definitive diagnosis rates of between 86-100% in unselected patients [29-31].

Diagnostic laparoscopy is very important especially in patients with pelvic disease and suspected appendicitis. In cases of uncertain preoperative diagnosis in septic and unstable patients, laparoscopy can shorten the observation period and avoid the need for imaging test [27].

## Source control

Source control encompasses all measures undertaken to eliminate the source of infection and to control ongoing contamination.

The most common source of infection in community acquired intra-abdominal infections is the appendix, followed by the colon, and then the stomach. Dehiscences complicate 5-10% of intra-abdominal bowel anastomoses, and are associated with a mortality increase [3].

Timing and adequacy of source control are the most important issues in the management of intra-abdominal infections, because inadequate and late operation may have a negative effect on the outcome.

Early control of the septic source can be achieved either by nonoperative or operative means.

Nonoperative interventional procedures include percutaneous drainages of abscesses.

Ultrasound and CT guided percutaneous drainage of abdominal and extraperitoneal abscesses in selected patients are safe and effective. Numerous studies in the surgery and radiology literature have documented the effectiveness of percutaneous drainage in selected patients, with cure rates of 62%-91% and with morbidity and mortality rates equivalent to those of surgical drainage [32-39].

The principal cause for failure of percutaneous drainage is misdiagnosis of the magnitude, extent, complexity, location of the abscess [40].

Surgery is the most important therapeutic measure to control intra-abdominal infections.

Generally, the choice of the procedure depends on the anatomical source of infection, on the degree of peritoneal inflammation, on the generalized septic response and on the patient's general conditions.

Surgical source control entails resection or suture of a diseased or perforated viscus (e.g. diverticular perforation, gastroduodenal perforation), removal of the infected organ (e.g. appendix, gall bladder), debridement of necrotic tissue, resection of ischemic bowel and repair/resection of traumatic perforations.

Laparotomy is usually performed through a midline incision.

The objectives are both to establish the cause of peritonitis and to control the origin of sepsis.

### Appendicitis

Acute appendicitis is the most common intra-abdominal condition requiring emergency surgery.

Acute appendicitis is the most common intra-abdominal condition requiring emergency surgery. Studies have demonstrated that antibiotics alone may be useful to treat patients with early, non perforated appendicitis, even if there is a risk of recurrence [41].

In 1995, Eriksson and Granstrom [42] published the results of a randomized trial of antibiotics versus surgery in the treatment of appendicitis. All patients treated conservatively were discharged within 2 days, except one who required surgery because of peritonitis secondary to perforated appendicitis. 14% of patients treated conservatively were readmitted within 1 year as a result of recurrent appendicitis and underwent surgery, when appendicitis was confirmed.

In 2006 Styrud et al. [43] published the results of a Swedish multicenter randomized trial. In the antibiotic group 86% improved without surgery; a rate of 14% of patients was operated on within 24 hours, and the diagnosis of acute appendicitis was confirmed in all but one patient, and he was suffering from terminal ileitis; 5% of patients had a perforated appendix in this group. The recurrence rate of symptoms of appendicitis among the patients treated with antibiotics was 14% during the 1-year follow-up.

Recently a further randomized clinical trial by Hanson et al. [44] compared antibiotic therapy versus appendectomy as primary treatment of acute appendicitis. Treatment efficacy was 90.8% for antibiotic therapy and 89.2 per cent for surgery. Recurrent appendicitis occurred in 13.9% of patients treated conservatively after a median of 1 year.

Although antibiotics may be used as primary treatment for selected patients with suspected uncomplicated appendicitis, appendectomy is still the gold standard therapy for acute appendicitis.

The advent of minimally invasive surgery has modified the surgical treatment of acute appendicitis and a lot of prospective randomized studies, meta-analyses, and systematic critical reviews have been published on the topic of laparoscopic appendectomy. Laparoscopic appendectomy is safe and effective, but open surgery still conferres benefits, in particular with regards to the likelihood of postoperative intra-abdominal abscess.

In 2007 a meta-analysis of 34 studies comparing laparoscopic appendectomy with open appendectomy was published by Bennett et al. [45]. The meta-analysis confirmed the findings of fewer surgical site infections and shorter hospitalization with laparoscopic appendectomy. Intra-abdominal abscesses were more common with laparoscopic appendectomy.

Although appendix abscess occurs in 10% of patients with acute appendicitis, its surgical management is surrounded with controversy. The traditional management of appendiceal mass has been initial conservative treatment followed by interval appendicectomy. Recently interval appendicectomy has been questioned, and there is much controversy whether interval appendicectomy is appropriate for adults with an appendiceal abscess. The main debate is based on the recurrence rate, the complication rate of interval appendicectomy, and the potential for underlying malignancy [46]. The results of a review by Andersonn and Petzold [47], based mainly on retrospective studies, supported the practice of nonsurgical treatment without interval appendectomy in patients with appendiceal abscess or phlegmon.

In 2007 another review [48] on management of appendiceal mass demonstrated that conservative management approach was successful in the majority of patients presenting with an appendix mass. The Authors concluded that after initial successful conservative management, routine use of interval appendicectomy was not justified in asymptomatic patients.

### Diverticulitis

Sigmoid diverticulitis is a common disease of the Western World and results in a significant number of hospital admissions.

Antibiotics are the standard of care for uncomplicated diverticulitis.

Percutaneous drainage is the intervention of choice for simple uniloculated abscesses. It has a success rate of more than 80%, but it may have a high failure rate in cases of complex multiloculated or inaccessible abscesses [49].

The use of antibiotics and percutaneous drainage in the management of diverticular abscesses facilitates single stage operation to perform subsequently an elective sigmoidectomy.

Ambrosetti et al. [50] studied retrospectively 73 patients with diverticular abscesses with a follow up of 43 months and found that 59% of the patients needed surgery either during the acute admission or as an elective procedure. The other patients did not need surgical intervention after conservative treatment either with or without percutaneous drainage. The study also compared the mesocolic abscesses with the pelvic ones. Pelvic abscesses exhibited an aggressive behaviour and therefore needed to be rapidly drained percutaneously and were likely to require surgery.

Brandt et al. [51] retrospectively compared patients with CT confirmed abscesses, treated by antibiotics alone and patient treated by antibiotics with percutaneous drainage. The patients treated with antibiotics alone achieved an outcome similar to patients treated with percutaneous drainage. The average abscess size was 4 cm in the antibiotic only group and 6 cm in percutaneous group. Failure rate of percutaneous drainage in this series was 33%.

Siewert et al. [52] reported that antibiotics alone were effective in resolving acute symptoms for abscess size less than 3 cm.

Urgent surgery for colonic diverticula perforations is indicated in patients with large or/and multiloculated diverticular abscesses inaccessible to percutaneous drainage or in whom clinical symptoms persist after CT guided percutaneous drainage, diverticulitis associated with free perforation and purulent or fecal diffuse peritonitis.

There is still controversy about the optimal surgical management of colonic diverticular disease, complicated by peritonitis.

Hartmann's resection has been considered the procedure of choice in patients with generalized peritonitis and remains a safe technique for emergency colectomy in perforated diverticulitis, especially in elderly patients with multiple co-morbidities [53]. More recently, some reports have suggested that primary resection and anastomosis is the preferred approach to diverticulitis, even in the presence of diffuse peritonitis [54,55].

In 2006 a sistematic review by Constantinides et al. [56] about primary resection with anastomosis vs. Hartmann's procedure in nonelective surgery for acute colonic diverticulitis was published. Patients selected for primary resection and anastomosis had a lower mortality than those treated with Hartmann's procedure in the emergency setting and comparable mortality under conditions of generalized peritonitis. This analysis highlighted the need for high-quality randomized trials comparing the two techniques.

Emergency laparoscopic resection in complicated diverticular disease is feasible and safe and may be performed by expert surgeons without additional morbidity and mortality [57,58]. In 2009 a randomized multicenter trial on laparoscopic sigmoid resection for diverticulitis was published [59]. In this trial patients with symptomatic diverticulitis of the sigmoid colon were randomized to either laparoscopic sigmoid resections or open sigmoid resections. The laparoscopic sigmoid resection was associated with a 15.4% reduction in major complication rates, less pain, improved quality of life, and shorter hospitalization at the cost of a longer operating time.

In high risk patients, a laparoscopic approach may be used for exploration and peritoneal lavage and drainage [60,61].

### Gastroduodenal perforations

Gastroduodenal perforations have decreased significantly in the last years thanks to the widespread adoption of medical therapies for peptic ulcer disease and stress ulcer prophylaxis among critically ill patients. Successful laparoscopic repair of perforated gastric and duodenal ulcers has been reported but the technique has yet to be universally accepted [62].

A systematic review was published in 2005 [63] in order to measure the effect of laparoscopic surgical treatment versus open surgical treatment in patients with a diagnosis of perforated peptic ulcer. Two randomised clinical trials, which were of acceptable quality, were included. No statistically significant differences between laparoscopic and open surgery in the proportion of abdominal septic complications, pulmonary complications or actual number of septic abdominal complications were found. With the information provided by the available clinical trials, laparoscopic surgery results were not clinically different from those of open surgery. This systematic review suggested that it was necessary to develop more randomised controlled trials with a greater number of patients.

The spontaneous perforation of gastric cancer is a rare fatal complication, occurring in 1% of patients with gastric cancer, and it has a wide hospital mortality range (0-82%). It has been also reported that about 10-16% of all gastric perforations are caused by gastric carcinoma [64]. In order to evaluate the gastric perforations and improve an alternative pathway for the management of this disorder without an available pathologist a study was realized by Ergul et al. [64]. The Authors recorded 513 patients who had undergone surgical treatment for gastric perforation due to gastric ulcus or gastric carcinoma in two medical centers. According to the results of their analysis patients age more than 60 years, an ulcus diameter (with edema) more than 6 cm, a perforation diameter more than 0.5 cm, a symptom duration of more than 20 h, and a white blood cell count less than 15.10(3)/microL, suggested for a gastric carcinoma. This system had a specificity of 98.7%, a sensitivity of 53.7%, a negative predicted value of 93.4%, and positive predicted value of 85.7%. They concluded that diagnosis of malignancy was often made only on postoperative or operative frozen pathologic examination. They suggested a new pathway for the gastric perforations, if a pathologist was not available during the operation.

### Small bowel perforations

Small bowel perforations are a less common source of peritonitis in the Western countries than the Eastern ones. Most small intestinal perforations are due to unrecognized intestinal ischemia. Treatment is most commonly resection of the involved segment. Small bowel obstruction has previously been considered a relative contraindication for laparoscopic management. A literature search of the Medline database by Ghosheh et al. [65] defined the outcome of laparoscopy for acute small bowel obstruction. Nineteen studies from between 1994 and 2005 were identified. The most common etiologies of obstruction were adhesions (83.2%), abdominal wall hernia (3.1%), malignancy (2.9%), internal hernia (1.9%), and bezoars (0.8%). Laparoscopic treatment was possible in 705 cases with a conversion rate to open surgery of 33.5%. Causes of conversion were dense adhesions (27.7%), the need for bowel resection (23.1%), unidentified etiology (13.0%), iatrogenic injury (10.2%), malignancy (7.4%), inadequate visualization (4.2%), hernia (3.2%), and other causes (11.1%). Morbidity was 15.5% (152/981) and mortality was 1.5% (16/1046). There were 45 reported recognized intraoperative enterotomies (6.5%), but less than half resulted in conversion. The Authors concluded that laparoscopy was an effective procedure for the treatment of acute small bowel obstruction with acceptable risk of morbidity and early recurrence

In eastern countries small bowel perforations usually arise on a background of enteric fever. These typhoid ileal perforations have a mortality rate up to 60% [66]. Early surgery is associated with a better outcome. A lot of surgical procedures have been described in these perforations such as simple closure, wedge excision or segmental resection and anastomosis, ileostomy and side to side ileo-transverse anastomosis after primary repair of the perforation [66].

Also primary intestinal tuberculosis is uncommon in European and North American countries and more common in Eastern countries. Most common site of extra pulmonary tuberculosis is the ileocaecal region and terminal ileum [67]. The most common complication of small bowel tuberculosis is obstruction due to the narrowing of the lumen by hyper plastic ileocaecal tuberculosis or stricture of small intestine and perforation in ulcerative type of tuberculosis, which are commonly multiple. Treatment of tubercular perforation of ileum depends upon the condition of the gut, general condition of the patient and number of perforation. Because of high mortality rate, the resection of the affected area and anastomosis may be the treatment of choice rather than primary closure [68].

### Cholecystitis

Laparoscopic cholecystectomy versus open cholecystectomy question has been extensively investigated. Beginning in the early 1990s, techniques and indications for laparoscopic management of the acutely inflamed gallbladder were discussed and laparoscopic cholecystectomy is now accepted as being safe for acute cholecystitis. Compared with delayed laparoscopic cholecystectomy, early laparoscopic cholecystectomy for acute cholecystitis is safer and shows lower rates of conversions than delay laparoscopic cholecystectomy. Several studies showed that early laparoscopic cholecystectomy resulted in a significantly reduced length of stay, no major complications, and no significant difference in conversion rates when compared with initial antibiotic treatment and delayed laparoscopic cholecystectomy [69-72].

In 2009 a prospective trial by González-Rodríguez et al. [73] about early or delayed laparoscopic cholecystectomy in acute cholecystitis confirmed that there is no advantage in delaying cholecystectomy for acute cholecystitis on the basis of complications, rate of conversion to open surgery, and mean hospital stay. Thus, early cholecystectomy should be the preferred surgical approach for patients with acute lithiasic cholecystitis.

Despite the evidence, early laparoscopic cholecystectomy is not the most common treatment for acute cholecystitis in practise and wrongly it remains common practice to treat acute cholecystitis with intravenous antibiotic therapy and interval laparoscopic cholecystectomy preferentially [74].

### Surgical options in patients with severe intra-abdominal infections

Patients with severe sepsis or septic shock may be complicated by high mortality rates. They may benefit of aggressive surgical treatment to control multiple organ dysfunction syndrome caused by ongoing intra-abdominal infection.

The surgical treatment strategies following an initial emergency laparotomy may include either a relaparotomy, only when the patient's condition demands it ("relaparotomy on-demand"), or a planned relaparotomy after 36-48 hours with temporarily abdomen closure or open abdomen.

The aim in the on-demand laparotomy is to perform reoperation only in those patients who may benefit from it.

The selection of the patients for relaparotomy is difficult and is based on clinical judgments with individual variability among surgeons. Currently, there is no consensus on which criteria may be used to undergo relaparotomy [75-80]

In order to determine which variables surgeons considered important in their decisional process of patient selection for relaparotomy Van Ruler et al. [75] published in 2008 the results of a questionnaire. Surgeons were asked to rank the importance of 21 clinical variables on their decision whether or not to reoperate in a patient with secondary peritonitis. Of variables labeled important only, a diffuse extent of abdominal contamination, localization of the infectious focus (upper gastrointestinal tract including small bowel), and both low and high leukocyte counts independently predicted positive relaparotomy. These variables had only moderate predictive accuracy. The results of the questionnaire demonstrated that there was no consensus among surgeons which variables were important in decision making for relaparotomy.

Over the past years, also Procalcitonin (PCT) was investigated as a laboratory variable to select patients for relaparotomy. Recently a study by Novotny et al. [81] evaluated procalcitonin (PCT) as a parameter for early detection of progressing sepsis after operative treatment of the infective source. PCT ratio appeared to be a valuable aid in deciding if further relaparotomies were necessary after initial operative treatment of an intraabdominal septic focus.

The final decision to perform a reoperation on a patient in the on-demand setting is generally based on patients generalized septic response and lack of clinical improvement.

The aim in the planned laparotomy is to perform every 36 to 48 hours inspection, drainage, and peritoneal lavage of the abdominal cavity. It is performed either with temporarily abdomen closure or open abdomen.

Surgical approach that leaves the abdomen open may both facilitate reexploration and prevent deleterious effects of abdominal compartment syndrome (ACS) [82].

In septic shock fluids infusion during resuscitation and their accumulation, bowel edema, and forced closure of the abdominal wall cause intra-abdominal hypertension (IAH) and consequently modify pulmonary, cardiovascular, renal, splanchnic, and central nervous system physiology causing significant morbidity and mortality.

Open treatment was introduced for the management of severe intra-abdominal infection and pancreatic necrosis some years ago [83]. However, severe complications such as evisceration, fistula formation, and the development of giant incisional hernias were observed. Therefore, the technique of open treatment was modified, leading to the concept of "covered laparostomy" [84-86]. Temporary closure of the abdomen may be achieved using gauze and large, impermeable, self-adhesive membrane dressings, absorbable meshes, nonabsorbable meshes, zippers and vacuum-assisted closure (VAC) devices. Vacuum-assisted fascial closure (VAC) has become an option for the treatment of open abdomen [87-90].

Some studies described open abdomen approach in the patients with severe sepsis or septic shock [91-94].

Some studies have indicated that the planned strategy increases the risk of multiple organ failure because it amplifies the systemic inflammatory response by multiple surgical lavages, leading to increased mortality [95,96], morbidity, ICU stays, and hospital stays [97].

In 2007 van Ruler et al. [98] published a randomized, clinical trial comparing on-Demand vs Planned Relaparotomy strategy in patients with severe peritonitis. The patients in the on-demand relaparotomy group did not have a significantly lower rate of death or major peritonitis-related morbidity compared with the planned relaparotomy group but did have a substantial reduction in relaparotomies, health care utilization, and medical costs.

In 2007 a randomised study by Robledo et al. [99] compared open with closed "on demand" management of severe peritonitis. The study however was interrupted after the inclusion of 40 patients because of a high rate of mortality for the open abdomen group (55 *vs *30%). The "open abdomen" was managed with only a non-absorbable polypropylene mesh.

## Antimicrobial therapy in Intra-abdominal Infections

Antimicrobial therapy plays an integral role in the management of intra-abdominal infections. The choice of an inadequate antimicrobial agent is a cause of therapeutic failure.

Complicated intra-abdominal infections are predominantly related to bowel perforation and contamination with its flora. The microbial etiology depends on the level of disruption of the gastrointestinal tract.

### Microbiology

The upper gastrointestinal tract (stomach, duodenum, jejunum, and upper ileum) contains relatively few microorganisms, less than 103 to 105 bacteria/mL.

Infections derived from the stomach, duodenum, and proximal small bowel can be caused by gram-positive and gram-negative aerobic and facultative organisms. The lower gastrointestinal tract contains hundreds of bacterial species, and concentrations of 1011-13 bacteria/mL.

Infections derived from distal ileum perforations can be caused by gram-negative facultative and aerobic organisms with variable density.

Colon-derived intra-abdominal infections can be caused by facultative and obligate anaerobic organisms, gram-negative facultative organism (Enterobacteriaceae with E. coli at the first place), other gram-negative bacilli and Enterococci.

Anaerobic bacteria are 1000 times more common than aerobes. With the exception of Bacteroides spp., most other anaerobes are the main barrier against colonization and infection by other pathogens.

The medical antecedents of the patient can affect the normal flora. In particular, patients hospitalised may be colonized by altered flora including multidrug-resistant nosocomial pathogens or *Candida spp*.

### Microbiological specimens

Once the diagnosis of complicated intra-abdominal infection is suspected, it is appropriate to begin empiric antimicrobial therapy before an exact diagnosis is established and before results of appropriate cultures are available.

The role of microbiologic workup of infected fluid has been debated in the last years.

Since the causative pathogens can easily be predicted in community acquired infections, bacteriological diagnosis is not necessary. The absence of impact of bacteriological cultures has been well documented, especially in appendicitis as the etiologic agents causing peritonitis are highly predictable and effective broad-spectrum antibiotic therapy is available.

In a prospective study, Gladman et al. [100] followed 721 consecutive appendicectomies. Swabs were performed in 463 cases. The culture was positive in 113 with the identification of 11 resistant microorganisms. Overall, 39 patients (5%) developed significant post-operative infective complications. Neither the presence of a positive intra-operative culture, nor the isolation of resistant organisms were significant in predicting infective complications. The authors concluded that the results of intra-operative culture did not influence clinical outcome in patients undergoing appendicectomy. The practice of taking routine microbiological swabs for culture had to be seriously questioned in patients undergoing appendicectomy

For higher-risk patients, cultures from the site of infection should be always obtained, Cultures should be performed from 1 specimen, provided it is of sufficient volume (at least 1 mL of fluid or tissue, preferably more). It should be transported to the laboratory in an appropriate transport system.

### Antimicrobial prophylaxis

Routine use of antimicrobial therapy is not appropriate for all patients with intra-abdominal infections.

In uncomplicated IAIs, when the focus of infection is treated effectively by surgical excision of the involved tissue, the administration of antibiotics is unnecessary beyond prophylaxis. Patients with an infected focus that can be eradicated effectively by surgical intervention can potentially be treated only with 24 hours antimicrobial prophylaxis. Antimicrobial prophylactic agents are indicated for patients with acute unperforated appendicitis or cholecystitis that are surgically removed [101].

Antibiotic prophylaxis is also sufficient for the patients with bowel necrosis due to a vascular accident or strangulating bowel obstruction, in whom there is no evidence of perforation or infected peritoneal fluid, for those with gastroduodenal perforations operated within 24 hours in the absence of antacid therapy or malignant disease, and for those with traumatic or iatrogenic bowel injury repaired within 12 hours [101].

### Risk stratification

Patients with intra-abdominal infections are generally classified into low risk and high risk.

The definition of "risk" in intra-abdominal infections remains vague. "High risk" is generally intended to describe patients with a high risk for treatment failure. In these patients intra-abdominal infections may be associated with a high risk of isolation of resistant pathogens from the intra-abdominal source. Effective management of high risk patients requires the early use of appropriate, broad-spectrum empirical antimicrobial therapy.

The stratification of the patient's risk is important to optimize the antibiotic treatment plan.

The increased mortality associated with inappropriate empiric antibiotic therapy cannot be reversed by subsequent modifications. Therefore knowledge of patient's risk is essential to begin treatment as soon as possible with the most appropriate regimen.

Many factors can contribute to a patient's risk for isolation of resistant pathogens. These include [102,103]:

• Health care-associated infections

• High severity of illness (APACHE II score >15)

• Advanced age

• Comorbidity and degree of organ dysfunction

• Poor nutritional status and low albumin level

• Immunodepression

• Presence of malignancy

In high risk patients the normal flora may be modified and intra-abdominal infections may be caused by several unexpected pathogens and by more resistant flora, which may include, methicillin-resistant *Staphylococcus aureus*, Enterococci, *Pseudomonas aeruginosa*, extended-spectrum β-lactamases producing *Enterobacteriaceae *(ESBLs) and *Candida *spp. In these infections antimicrobial regimens with broader spectrum of activity are recommended, because adequate empirical therapy appears to be important in reducing mortality.

Health care-associated infections are commonly caused by more resistant flora, and for these infections, complex multidrug regimens are always recommended. Although transmission of multidrug resistant organisms is most frequently documented in acute care facilities, all healthcare settings are affected by the emergence and transmission of antimicrobial-resistant microbes.

Among intra-abdominal infections post-operative peritonitis is a life-threatening infection and carries a high risk of complications and mortality.

In order to describe the clinical, microbiological and resistance profiles of community-acquired and nosocomial intra-abdominal infections a prospective, observational study (EBIIA) [104] was completed in French. The results or this study were published in 2009. From January to July 2005, patients undergoing surgery/interventional drainage for IAIs with a positive microbiological culture were included by 25 French centres. The principal results of EBIIA were a higher diversity of microorganisms isolated in nosocomial infections and decreased susceptibility among these strains.

In order to assess the microbiological differences, particularly with respect to the type of bacteria recovered and the level of antimicrobial susceptibility between community-acquired and nosocomial IAIs, the results of an interesting prospective observational study were published by Seguin et al. [105] in 2006. Community-acquired peritonitis accounted for 44 cases and nosocomial peritonitis for 49 cases (post-operative in 35 cases). In univariate analysis, the presence of MDR bacteria was associated significantly with preoperative and total hospital lengths of stay, previous use of antimicrobial therapy, and post-operative antimicrobial therapy duration and modifications. A 5-day cut-off in length of hospital stay had the best specificity (58%) and sensitivity (93%) for predicting whether MDR bacteria were present. In multivariate analysis, only a composite variable associating pre-operative hospital length of stay and previous use of antimicrobial therapy was a significant independent risk-factor for infection with MDR bacteria. The Authors concluded that the knowledge of these two factors might provide a more rational basis for selecting initial antimicrobial therapy for patients with complicated intra-abdominal infections. In order to investigate patient characteristics associated with a high risk of isolation of resistant pathogens from an intra-abdominal source, the results of a retrospective study by Swenson et al. [106] were published recently. Complicated intra-abdominal and abdominal organ/space surgical site infections treated over a ten-year period in a single hospital were studied. A total of 2,049 intra-abdominal infections were treated during the period of study, of which 1,182 had valid microbiological data. Health care association, corticosteroid use, organ transplantation, liver disease, pulmonary disease, and a duodenal source all were associated with resistant pathogens.

Low risk patients are generally those with community-acquired infections without risk factors. Intra-abdominal infections in low risk patients are associated with expected pathogens with known susceptibilities. Empirical agents in these patients must be directed at providing reliable activity against E coli, other gram negative facultative bacteria, and B fragilis. Antibiotic regimens with a broader spectrum of activity are not recommended for low risk patients with intra-abdominal infections, because such regimens may carry a greater risk of toxicity and facilitate acquisition of more resistant organisms.

### Antimicrobial regimens

Intra-abdominal infections may be managed with either single or multiple antimicrobial regimens.

Recently the new guidelines for the management of complicated intra-abdominal infections by the Surgical Infection Society and the Infectious Diseases Society of America were published [103].

According to the guidelines, for adults with extra-biliary mild-to-moderate severity community acquired complicated infections, the use of ticarcillin-clavulanate, cefoxitin, ertapenem, moxifloxacin, or tigecycline as single-agent therapy or combinations of metronidazole with cefazolin, cefuroxime, ceftriaxone, cefotaxime, levofloxacin, or ciprofloxacin are recommended [103].

For adults with extra-biliary high severity complicated infections, meropenem, imipenem-cilastatin, doripenem, piperacillin/tazobactam, ciprofloxacin or levofloxacin in combination with metronidazole, or ceftazidime or cefepime in combination with metronidazole are recommended. Because of increasing resistance of *Escherichia coli *to fluoroquinolones, local population susceptibility profiles and, if available, isolate susceptibility should be always reviewed [103].

Selection of specific antimicrobial therapy for pediatric patients with complicated intra-abdominal infection should be based on the origin of infection (community vs health care), severity of illness, and safety of the antimicrobial agents in specific pediatric age groups. For pediatric patients with complicated intra-abdominal infection, ertapenem, meropenem, imipenem/cilastatin, ticarcillin-clavulanate, and piperacillin-tazobactam as single-agent therapy or Ceftriaxone, cefotaxime, cefepime, ceftazidime, each in combination with metronidazole, gentamicin or tobramycin, each in combination with metronidazole or clindamycin, and with or without ampicillin are recommended [103].

Beta-lactam/beta-lactamase inhibitor combinations, have been widely used in the last decade. Their in vitro activity includes gram-positive (include Enterococci in their spectrum), gram-negative and anaerobe organisms [107,108].

Among beta-lactam/beta-lactamase inhibitor agents, ticarcillin/clavulanate and ampicillin/sulbactam have been used in the treatment of intra mild to moderate intra-abdominal infections. Ampicillin-sulbactam is still indicated for community infections of mild-to-moderate severity [109], however the increasing resistance of *Enterobacteriaceae *reported in the last decade could compromise its clinical effectiveness [110].

Piperacillin/tazobactam is a beta-lactam/beta-lactamase inhibitor combination with increased gram-negative spectrum and anti-pseudomonas activity. Piperacillin/tazobactam retains in vitro activity against broad-spectrum beta-lactamase-producing, many extended-spectrum beta-lactamase-producing Enterobacteriaceae and many *Pseudomonas *isolates. It is still a reliable option for the empiric treatment of high risk intra-abdominal infections [111].

Carbapenems have a spectrum of antimicrobial activity that includes Gram-positive (except resistant gram positive cocci) and Gram-negative aerobic and anaerobic pathogens.

Group 1 carbapenems includes ertapenem, a once a day carbapenem that shares the activity of imipenem and meropenem against most species, including extended-spectrum β-lactamase (ESBL)-producing pathogens [112,113], but is not active against non-fermentative gram negative and *Enterococcus*. Ertapenem is particularly suitable for low risk community-acquired intra-abdominal infections. Once-daily ertapenem is an interesting option for the treatment of these infections.

Group 2 includes imipenem/cilastatin, meropenem and doripenem, that share activity against non-fermentative gram-negative bacilli and are particularly suitable for severe infections.

Doripenem is a new 1-β-methyl carbapenem recently approved by the Food and Drug Administration for the treatment of complicated intra-abdominal infections and complicated urinary tract infections. Doripenem similarly to imipenem and meropenem, has a broad-spectrum activity against Gram-positive, Gram-negative, and anaerobic bacteria [114,115]. Doripenem is more effective, in vitro, than meropenem and imipenem against Pseudomonas aeruginosa [116,117].

Group 2 carbapenems are important options for the empirical treatment of high risk intra-abdominal infections also in hospitalized patients.

In the past Cephalosporins have been often used in the treatment of intra-abdominal infections. Cephalosporins except, the second generation subgroup with activity against Bacteroides spp (cefoxitin and cefotetan), do not exhibit anti-anaerobic activity and must always be used in combination with anti-anaerobic agents [118].

Second-generation cephalosporins are widely used in surgical prophylaxis and trauma. They have been used in the treatment of mild-to-moderate community-acquired infections, but limitations in their spectra and microbial resistance restrict their utility in complicated intra-abdominal infections.

Among third generation cephalosporins both subgroups with poor activity against Pseudomonas aeruginosa (cefotaxime, ceftriaxone, and ceftizoxime) and with good activity against Pseudomonas aeruginosa (cefoperazone and ceftazidime) have been used in the treatment of intra-abdominal infections in association with metronidazole. Both cephalosporins acquired resistance in enterobacteriaceae [119,120] and intrinsic resistance in Enterococci [121] may limit cephalosporins use in high risk intra-abdominal infections especially in healt-care infections.

Cefepime is a 'fourth-generation' cephalosporin. It was introduced into clinical practice in 1994 and is used in association with metronidazole for the treatment of severe infections [122]. Cefepime possesses higher in vitro activity than other extended-spectrum cephalosporins against common Gram-negative and Gram-positive pathogens and may be effective, in association with metronidazole, in high risk intra-abdominal infections [103,123]. The results of a meta-analysis by Yahav et al. [124] in 2007 indicated a potential increased mortality in patients treated with cefepime compared with patients treated with other β-lactam drugs. Caution in the use of cefepime should be adopted until new evidence on cefepime safety is available [125].

Fluoroquinolones have been widely used in the last years for the treatment of intra-abdominal infections, because of their excellent activity against aerobic Gram-negative bacteria and tissue penetration. In addition all the fluoroquinolones are rapidly and almost completely absorbed from the gastrointestinal tract. Peak serum concentrations obtained after oral administration are very near those achieved with intravenous administration [126].

Quinolones do not exhibit potent antianaerobic activity and have been used in combination with other therapeutic antianaerobic agents. Many studies have proved fluoroquinolones in association with metronidazole an effective therapeutic option for the treatment of patients with intra-abdominal infections since their discovery [127]. The combination of ciprofloxacin/metronidazole has been one of the most commonly used regimens for the treatment of patients with severe complicated intra-abdomianl infections in the last years. Ciprofloxacin is a potential therapeutic option for the treatment of infections caused by Pseudomonas and ESBL producing enterobacteriaceae; however, in recent years, the consumption of ciprofloxacin has risen and ESBL-producing isolates resistant to fluoroquinolones has increased over time, initially in *K. pneumoniae *and later also in *E. coli *[128]. Besides ciprofloxacin has unreliable activity against Enterococci and staphylococci. Nowadays doubts emerge about the advisability of using ciprofloxacin plus metronidazole to treat severe intra-abdominal infections in high risk patients.

Moxifloxacin has shown activity against a wide range of aerobic Gram-positive and Gram-negative [129]. Compared with ciprofloxacin, moxifloxacin has enhanced activity against Gram-positive bacteria with a decrease in activity against Gram-negative bacteria (Enterobacteriaceae and Pseudomonas species) [130].

Among quinolones moxifloxacin seems to be effective also against *Bacterioides fragilis*, suggesting that it may be effective for the treatment of low risk intra-abdominal infections without antianaerobic agents [131-133].

Levofloxacin has a spectrum of activity similar to moxifloxacin's, and even if compared to moxifloxacin it has no activity against anaerobic bacteria, less activity against resistant Gram Positive bacteria [134], it has a potential activity against *Pseudomonas *[135]. In association with metronidazole it is effective for the treatment of low risk intra-abdominal infections.

Aminoglycosides such as gentamicin, tobramycin and amikacin are particularly active against aerobic Gram-negative bacteria and act synergistically against certain Gram-positive organisms. Gentamicin is the most commonly used aminoglycoside, but amikacin may be particularly effective against resistant organisms. They are effective against Pseudomonas aeruginosa. Aminoglycosides are not effective against anaerobic bacteria. Because of ototoxicity and nephrotoxicity aminoglycosides have not often been recommended for the routine empiric treatment of community-acquired intra-abdominal infections [103]. Aminoglycosides may be reserved for patients with allergies to b-lactam agents and may be selected for treatment of patients with health care-associated intra-abdominal infection, depending on local susceptibility patterns of nosocomial gram-negative bacilli [103].

Aztreonam is a parenteral synthetic beta-lactam antibiotic and the first monobactam to be marketed. Aztreonam exhibits potent and specific activity in vitro against a wide spectrum of Gram-negative aerobic pathogens including *Pseudomonas aeruginosa*. It has no useful activity against Gram-positive bacteria or anaerobes, but has very broad spectrum against Gram-negative aerobes, including *Pseudomonas aeruginosa *[136]. In the treatment of complicated intra-abdominal infections it is not practical as a single agent since anaerobic and Gram-positive bacteria are not susceptible to aztreonam [137].

Tigecycline is the first representative of the glycylcycline class of antibacterial agents to be marketed for clinical use [138,139].

Tigecycline represents a new treatment option for complicated intra-abdominal infections due to its favourable in vitro activity against a wide variety of aerobic Gram-positive, (including multidrug-resistant pathogens such as MRSA, VISA, VRSA, VRE) [140], Gram-negative (including ESBL-producing strains of *E. coli *and *Klebsiella*) [141,142] and anaerobic organisms. Tigecycline has no activity in vitro against *P. aeruginosa *and *P. mirabilis*.

Tigecycline has showed also considerable, though not universally consistent, antimicrobial activity against MDR (including carbapenem-resistant) *Acinetobacter *spp [143-145].

Tigecycline is recommended by IDSA guidelines for empiric treatment of mild-to-moderate severity infections [103].

Tigecycline maintains satisfactory profiles of safety and efficacy in treatment of multidrug resistant bacteria, in complicated intra-abdominal infections. Judicious use of antibiotics for multidrug resistant pathogens is important to preserve their effectiveness, and tigecycline is one of the few available compounds active against multidrug resistant strains. It may be more suitable to use tigecycline for empiric or definitive treatment of patients with high risk intra-abdominal infections. Combinations with other broad-spectrum antibiotics may be suitable in critically ill patients or in patients with health-care infections known or suspected to be owing to *Pseudomonas aeruginosa*.

### Adequate therapy

Adequate indications and duration of therapy are particularly important. Inadequate duration of treatment is probably the main inappropriate use of antibiotics in surgical practice and the intensive care unit. Antimicrobial therapy for established infections should be continued until normalization of clinical signs of infection occurs, including normalization of temperature and WBC count. If clinical signs and symptoms persist after a reasonable course of antibiotic therapy, another infectious cause should be sought rather than prolonging antibiotic treatment for the initial infection.

Unnecessary broad coverage or prolonged therapy can carry high costs, toxicities of therapy and *Clostridium difficile *colitis superinfection. *Clostridium difficile *causes 15%-25% of all cases of antibiotic-associated diarrhea, the severity of which ranges from mild diarrhea to fulminant pseudomembranous colitis [146].

Over the past years, some Authors have investigated procalcitonin (PCT) to guide duration of antibiotic therapy. Currently, procalcitonin (PCT) has emerged as a laboratory variable that allows early differentiation between SIRS and sepsis. It was recently been used to guide antibiotic treatment in medical patients with pulmonary diseases [147].

Recently, Hochreiter et al. [148] published a prospective trial to value the role of procalcitonin for guiding antibiotic therapy in surgical intensive care patients. They enrolled a total of 110 surgical intensive care patients receiving antibiotic therapy after confirmed or high-grade suspected infections. In 57 patients antibiotic therapy was guided by daily PCT and clinical assessment and adjusted accordingly. The control group comprised 53 patients with a standardized duration of antibiotic therapy over eight days. In the PCT group the duration of antibiotic therapy was significantly shorter than in the control group without negative effects on clinical outcome.

Inappropriate antibiotic therapy of intra-abdominal infections may result in poor patient outcomes.

In order to value the association between inappropriate antibiotic therapy and clinical outcomes for complicated community-acquired intra-abdominal infections Tellado et al. [149] reviewed patient records from October 1998 to August 2002 in 24 hospitals in Spain. They classified initial empiric therapy as appropriate if all isolates were sensitive to at least 1 of the antibiotics administered. Inappropriate initial antibiotic therapy was associated with a significantly higher rate of unsuccessful outcomes including death, re-operation, re-hospitalization or additional parental antibiotic therapies.

In 2008 Edelsberg et al. [150] explored the economic consequences of failure of empiric therapy in antibiotic therapy in hospitalized adults with complicated intra-abdominal infection. Using a large U.S. multi-institutional database, they identified all hospitalized adults admitted between April 2003 and March 2004 with cIAI, who had undergone laparotomy, laparoscopy or percutaneous drainage and had received intravenous antibiotics. Antibiotic failure was considered on the basis of the need for reoperation or receipt of other antibiotics postoperatively. Among 6,056 patients who met the study entrance criteria, 22.4% failed initial antibiotic therapy. Failure of initial intravenous antibiotics in hospitalized adults with cIAIs was associated with longer hospitalization, higher hospital charges, and higher mortality rate.

### De escalation approach in critically ill patients

The rise in antibiotic resistance in the ICU poses serious problems for the management of critically ill patients. The choice of empiric antibiotic therapy can have a significant impact on patient outcome when resistant pathogens may be involved. Empiric antimicrobial therapy for patients with severe sepsis or septic shock may be ineffective if the responsible organism is not susceptible to available antibiotics. Therefore, attention has been focused on the need for strategies to combat antibiotic resistance in the ICU.

In critically ill patients a de escalation approach may be recommended. For years antibiotic therapy has been started with a basic agent and only once microbiological culture results and susceptibility tests were available, more potent compounds were used. The traditional approach, however, may no longer be appropriate for critically ill patients in the current era of increasing antibiotic resistance.

Rising resistance rates and better understanding of the inflammatory process prompted some experts to advocate initial therapy with broad-spectrum, initially in severe pneumonias [151,152].

This two-stage approach of using aggressive initial therapy followed by de-escalation allows serious infection to be treated immediately and effectively avoiding antibiotic overuse, potential resistance and excessive costs.

## Multidrug-resistant pathogens

The threat of antimicrobial resistance has been identified as one of the major challenges in the management of complicated intra-abdominal infections.

Over the past few decades, an increase of infections caused by antibiotic-resistant pathogens, including methicillin-resistant *Staphylococcus aureus*, vancomycin-resistant *Enterococcus *species, carbapenem-resistant *Pseudomonas aeruginosa*, extended-spectrum beta-lactamase-producing *Escherichia coli *and *Klebsiella spp*., and multidrug-resistant *Acinetobacter *spp., has been observed, also in intra-abdominal infections.

Management of severe intra-abdominal infections must always include a balance between optimizing empirical therapy, which has been shown to improve outcomes, and reducing unnecessary antimicrobial use. Bacterial resistance is becoming a very important problem. Despite increasing antimicrobial resistance and multi-drug resistance in clinical isolates, there are few novel antimicrobial agents in development. Some broad-spectrum agents maintain still satisfactory profiles of safety and efficacy in treatment of multidrug resistant bacteria in complicated intra-abdominal infections but they must be used judiciously to preserve their effectiveness against multidrug resistant pathogens.

### Enterococcus

Enterococcus infections are difficult to treat because of both intrinsic and acquired resistance to many antibiotics.

Enterococci are intrinsically resistant to many penicillins, and all cephalosporins with the possible exception of ceftobiprole and ceftaroline, currently undergoing clinical evaluation. Besides Enterococci have acquired resistance to many other classes of antibiotics, to which the organisms are not intrinsically resistant, including fluoroquinolones, aminoglycosides, and penicillins. Many strains of E. faecalis are susceptible to certain penicillins, carbapenems, and fluoroquinolones; however, virtually all strains of E. faecium are resistant to these agents [153].

Vancomycin-resistant Enterococci (VRE) infections have bee associated with increased morbidity and mortality [154,155]. Resistance of Enterococci to vancomycin was reported in Europe in 1986 and the prevalence of infections related to VRE has continued to increase annually [156].

Many factors can increase the risk of colonization with VRE. These include previous antibiotic therapy, the number and duration of antibiotics received, prolonged hospitalization, hospitalization in an intensive care unit and concomitant serious illness [157].

Several antibiotics have been implicated for VRE acquisition, but use of vancomycin and third-generation cephalosporins have appeared to be associated most commonly with the spread of VRE [158].

Against Vancomycin-resistant Enterococci (VRE) linezolid, tigecycline, quinupristin/dalfopristin, or daptomycin should be considered.

Empirical treatment against Enterococci and has not been generally recommended for patients who have community-acquired intra-abdominal infections [103]. However Enterococci isolation may be a risk factor for treatment failure and it has been suggested that if initial antibiotic therapy does not cover for Enterococci, patients may have an increased risk of postoperative complications and death [159,160]. Recently Riché et al. [161] published a prospective observational study involving 180 consecutive patients with secondary generalized peritonitis (community-acquired and postoperative) which analyzed clinical and bacteriological factors associated with the occurrence of shock and mortality in patients with secondary generalized peritonitis. Frequency of septic shock was 41% and overall mortality rate was 19%. Patients with septic shock had a mortality rate of 35%, versus 8% for patients without shock. Septic shock occurrence and mortality rate were not different between community-acquired and postoperative peritonitis. Age over 65, two or more microorganisms, or anaerobes in peritoneal fluid culture were independent risk factors of shock. Intraperitoneal yeasts and Enterococci were associated with septic shock in community-acquired peritonitis. Their findings supported the deleterious role of *Enterococcus *species in peritoneal fluid, reinforcing the need of prospective trials to evaluate systematic treatment against these microorganisms in patients with secondary peritonitis.

Enterococcal infection should be suspected in patients with post-operative or nosocomial infections, in patients with recent exposure to broad-spectrum antimicrobial agents especially cephalosporins, in immunocompromised patients and in patients with valvular heart disease or prosthetic intravascular materials [103]. Expanded spectrum agents against enterocci should be also recommended for these patients with severe sepsis and septic shock in which a de escalation approach of an initially broad antimicrobial regimen to scale when definitive culture results are available [162,163].

For community-acquired biliary infection, antimicrobial activity against enterococci should be not required, because the pathogenicity of enterococci has not been demonstrated. For selected immunosuppressed patients, particularly those with hepatic transplantation, enterococcal infections may be significant and require treatment also for community-acquired biliary infection [103].

### Methicillin resistant Staphylococcus aureus

Methicillin resistant *Staphylococcus aureus *(MRSA) is the other multiresistant Gram-positive nosocomial pathogen that causes severe morbidity and mortality worldwide.

Methicillin-resistant *S. aureus *(healthcare-acquired MRSA; HA-MRSA) isolates have been a source of serious infections in hospitals since the 1960s and in the period 1975 to 1991, the National Nosocomial Infection Surveillance data showed that within all hospitals, there was an increase from 2% to 29% in the proportion of methicillin resistance among S aureus, and an increase to 38% in those hospitals with more than 500 beds [164].

In recent years isolates of community-associated (CA-MRSA) have been identified too [165].

The traditional antibiotic therapy for MRSA has always been glycopeptides.

The widespread occurrence of MRSA induced an inevitable increase of vancomycin and teicoplanin use, causing a selective pressure to develop glycopeptides resistance so that in 1997 the first vancomycin-intermediate *Staphylococcus aureus *(VISA) was reported and after some years the first vancomycin-resistent *Staphylococcus aureus *(VRSA) was also documented [166].

Multiresistant *Staphylococcus aureus *diffusion highlights the importance of the development of new agents for the appropriate treatment of infections where highly resistant pathogens are suspected or known. The list of antimicrobial agents with activity against MRSA is short, including Quinupristin/dalfopristin, daptomycin, linezolid and tigecicline. Recently resistances also to linezolid were identified [167].

Empiric antimicrobial against MRSA should be provided to patients with health care-associated intra-abdominal infections who are known to be colonized with the organism or who are suspected of having an infection due to this organism because of prior treatment failure and significant antibiotic exposure [103].

### Extended-spectrum β-lactamases (ESBLs) producing Enterobacteriaceae

Over the past few years a notable increase in antibiotic resistance among Gram-negative bacteria recovered from hospitalized patients has been reported, especially in critically ill patients [168].

During the last decade, the emergence of multidrug-resistant (MDR) Gram-negative bacteria such as *Pseudomonas aeruginosa*, *Acinetobacter baumannii*, *Stenotrophomonas maltophilia *and extended-spectrum beta-lactamase (ESBL) producing *Enterobacteriaceae *has become a growing problem.

In the specific context of intra-abdominal infections, resistance to β-lactams, mediated by extended-spectrum β-lactamases (ESBLs) is a particular concern [169,170].

Acquired resistance to beta-lactams is mainly mediated by extended-spectrum beta-lactamases (ESBLs) that confer resistance to the penicillins, first-, second-, and third-generation cephalosporins, and aztreonam, and are inhibited by b-lactamase inhibitors. Extended-spectrum beta-lactamases (ESBLs) which are encoded by genes that can be exchanged between bacteria. Beta-lactamase genes are often associated with resistance determinants to non-beta-lactam agents such as aminoglycosides and fluoroquinolones, and strains producing ESBLs often exhibit complex multidrug resistant phenotypes and sometimes are panresistant [171,172]. Therefore, antibiotic options in the treatment of ESBL-producing organisms are extremely limited. Carbapenems are the drugs of choice for treatment of infections caused by ESBL-producing organisms in intra-abdominal infections even if, use of carbapenems has been associated with the emergence of carbapenem-resistant bacterial species [173]. Tigecycline has substantial antimicrobial activity against ESBL-producing Enterobacteriaceae but it merits further evaluation [141,142].

Data from SMART (Study for Monitoring Antimicrobial Resistance Trends) in the period 2005 to 2007 found that the most frequently isolated organisms were *Escherichia coli, Klebsiella pneumoniae *and *Enterobacter cloacae*, of which 18% of *E. coli *and 26.2% of *K. pneumoniae *were positive for extended-spectrum beta-lactamase (ESBL) [174]. Overall, resistance among ESBL-producing isolates increased during 2005-2007 and resistance rates in 2007 were generally higher than data from previous years. Carbapenems were the only agents that maintained consistent activity against ESBL-producing isolates. In such study Tigecycline was not tested.

High risk patients for ESBL producing organisms infection are often seriously ill patients with prolonged hospital stays in whom invasive medical devices are present [119]. Other risk factors have been found and include the presence of nasogastric tubes, gastrostomy or jejunostomy tubes and arterial lines, administration of total parenteral nutrition, recent surgery, hemodialysis, decubitus ulcers, and poor nutritional status [119].

There is a strong relationship between antibiotics and acquisition of an ESBL producing strain [119]. The antibiotic classes found to be associated with ESBL-producing organisms include especially cephalosporins and quinolones.

### Pseudomonas aeruginosa

Dramatic may be multidrug-resistant non fermenting Gram-negative bacteria in ICUs. *Pseudomonas aeruginosa *is among the leading pathogens causing nosocomial infections especially in the ICUs. *P. aeruginosa *resistance depends on the bacteria's intrinsic as well as remarkable ability to acquire antibiotic resistance [175,176].

Antimicrobial agents with reliable anti-pseudomonas activity that are commonly prescribed are limited to antipseudomonas carbapenems, piperacillin/tazobactam, ceftazidime, cefepime, fluoroquinolones, aminoglycosides, aztreonam.

In the treatment of the most problematic multidrug resistant *Pseudomonas *strains, the class of polymyxins, represented by polymyxin B and polymyxin E (colistin), has gained a principal role despite its high toxicity [177].

Data from SMART (Study for Monitoring Antimicrobial Resistance Trends) in the period 2005 to 2007 no antimicrobial agent exhibited susceptibility of >90% against *Pseudomonas*. The most active agents were amikacin and piperacillin/tazobactam to which 86,5% of Pseudomonas were susceptible.

No clear data or expert opinion are available, but P. aeruginosa coverage is generally recommended in patients with health-care intra-abdominal infections, even if in some populations and communities, a relatively and inexplicably high prevalence of *Pseudomonas aeruginosa*, may be associated with community-acquired appendicitis [178] and may impact the selection of appropriate empiric antibiotic therapy [103].

### Acinetobacter baumannii

Also *Acinetobacter baumannii *is increasingly reported as the cause of nosocomial infections. Acinetobacter isolates demonstrate increasing resistance to commonly prescribed antimicrobials. Multidrug-resistant Acinetobacter baumannii is one of the most difficult healthcare-associated infections to control and treat [179-181].

The management of *A*. *baumannii *infections is difficult, because of the increasing number of isolates exhibiting resistance to multiple classes of antibacterial agents [182,183]. Agents potentially effective against *A*. *baumannii *include carbapenems, aminoglycosides (amikacin or gentamicin), tetracyclines (minocycline or doxycycline) and sulbactam [184].

Data from TEST (The Tigecycline Evaluation and Surveillance Trial) during 2004-2007 showed that the most active agents against *Acinetobacter *spp. were tigecycline, minocycline and Group 2 carbapenems [185].

Resistance to tigecycline and carbapenems makes multidrug-resistant Acinetobacter infections difficult to treat. Colistin and polymyxin B have been used to treat highly resistant Acinetobacter infections. The choice of appropriate therapy is further complicated by the toxicity of colistin [186,187].

Acinetobacter isolates resistant to colistin and polymyxin B have also been reported [188]. Studies have demonstrated in-vitro susceptibility of multidrug-resistant Acinetobacter to various synergistic combinations of antimicrobials including carbapenems, colistin, rifampin, ampicillin-sulbactam and tigecycline [189,190].

### Bacteroides fragilis

The *Bacteroides fragilis *group is a predominant component of the normal bacterial flora of the gastrointestinal tract. These bacteria are frequently isolated from mixed aerobic-anaerobic infections, such as intra-abdominal infections.

The increasing resistance to antimicrobial agents among anaerobic pathogens has been a global problem in the last years. Susceptibility to antibiotics varies considerably among the species of the group.

Clinically, Bacteroides species have exhibited increasing resistance to many antibiotics. Resistance to the most active drugs, such as imipenem, piperacillin-tazobactam, and metronidazole, has been found in occasional strains [191,192].

Most clinical laboratories do not routinely determine the species of the organism or test the susceptibilities of any anaerobic isolates, including those in the *B. fragilis *group, because of technical difficulties surrounding *Bacteroides *susceptibility testing. Consequently, the treatment of anaerobic infections is selected empirically, based on published reports on patterns of susceptibility [193].

A multicenter study by Aldridge et al. [194] surveyed the susceptibilities of 556 clinical anaerobic isolates, predominantly intra-abdominal, from four large medical centers. Piperacillin-tazobactam was the only antimicrobial agent to which all the isolates were susceptible. Similarly, imipenem, meropenem, and metronidazole were highly active (resistance, <0.5%), whereas the lowest susceptibility rates were noted for ciprofloxacin, and clindamycin.

A recent multicenter study by Snydman et al. [193] determined the susceptibility trends for the species of the *Bacteroides fragilis *group against various antibiotics from 1997 to 2004 by using data for 5,225 isolates referred by 10 medical centers in the United States. Resistance to carbapenems was rarely seen in this study (<1.5%). The trends in resistance to piperacillin-tazobactam, ampicillin-sulbactam, and cefoxitin were species dependent. Resistance of *B. fragilis*, to clindamycin increased significantly, similar results were seen for moxifloxacin. Resistance rates for tigecycline were low and stable during the 5-year period during which this agent was studied.

### Candida

In the last years there has been a significant increase in the incidence of invasive infections due to *Candida *species. Candida intra-abdominal infections are associated with poor prognosis [195]. Thirty to forty percent of patients with recurrent gastrointestinal perforation/anastomotic leakage develop intra-abdominal invasive candidiasis [196].

The most frequently implicated risk factors include the use of broad-spectrum antibacterial agents, use of central venous catheters, receipt of parenteral nutrition, receipt of renal replacement therapy by patients in ICUs, neutropenia, and receipt of immunosuppressive agents (including glucocorticosteroids, chemotherapeutic agents, and immunomodulators). Patients with health care-associated intra-abdominal infection are at higher risk of *Candida *peritonitis, particularly patients with recurrent gastrointestinal perforations and surgically treated pancreatic infection.

Empiric antifungal therapy with fluconazole may decrease the incidence of *Candida *peritonitis in high-risk patients [103].

Fluconazole, is recommended as initial therapy [197]. An echinocandin (Caspofungin, Anidulafungin, or Micafungin) is preferred for patients with recent azole exposure, patients with moderately severe to severe illness, or patients who are at high risk of infection due to *C. glabrata *or *C. krusei*.

Avoiding unnecessary antibiotics and optimizing the administration of antimicrobial agents will help to improve patient outcomes and minimize further pressures for resistance.

Several strategies aim at achieving optimal use of antimicrobial agents, such as guidelines or protocols, restricting the hospital formulary, combining antibiotic therapy, antibiotic rotation, area-specific antimicrobial therapy, antimicrobial de-escalation and infections controls [198], but it is important that surgeons know antibiotic administration minimal requirements, such as antibiotics spectrum of activity and drug effective dosing. Without these minimal requirements surgeons will increase the likelihood of treatment failures and antibiotic resistance.

## Conclusions

Complicated intra-abdominal infections are an important cause of morbidity and are frequently associated with a poor prognosis.

Despite advances in diagnosis, surgery, antimicrobial therapy mortality associated with complicated intra-abdominal infections remains still unacceptably high.

Early adequate source control remains the cornerstone of intra-abdominal infection management. Early control of the septic source can be achieved either by nonoperative or operative means. Timing and adequacy of source control is the most important issue in the management of intra-abdominal infections, because an inadequate and late operation may have a negative effect on outcome.

Recent advances in interventional and more aggressive techniques are debated and are not validated by limited prospective trials.

Concomitant adequate empiric antimicrobial therapy further influences patients morbidity and mortality. Inappropriate antibiotic therapy of intra-abdominal infections may result in poor patient outcome and the selection of an appropriate agent is a real challenge because of the emerging resistance of target organisms to commonly prescribed antibiotics.

## Competing interests

The author declares that they have no competing interests.

## Authors' contributions

MS designed the study, performed literature search and manuscript preparation.
